# Managing Complex Wounds in Skilled Nursing Facilities (SNFs)

**DOI:** 10.7759/cureus.47581

**Published:** 2023-10-24

**Authors:** James Ebot

**Affiliations:** 1 Public Health, Johns Hopkins Bloomberg School of Public Health, Baltimore, USA

**Keywords:** advanced wound care, negative-pressure wound therapy, and wound care in long-term care settings, surgical wound complications, wound care management, post-surgical wounds

## Abstract

Wounds, especially chronic wounds, can be clinically challenging to manage. The presence of a chronic wound in a patient can not only cause nociceptive pain but also psychological and emotional pain. In extreme cases, they can be life-threatening if they present with infection and sepsis from poor wound care. This paper highlights the care of a patient in a skilled nursing facility who presented with a very complicated post-surgical abdominal wound, secondary to an infected post-surgical incision. The skilled nursing facility was very concerned about the size, depth, and nature of the wound, and talks were underway to transfer the patient to a different long-term acute care facility or to the hospital for more specialized care. Thanks to the weekly rounds of a specialized wound care physician to the facility, and a dedicated wound care nurse to execute the physician’s orders, the wound was adequately cared for and close to resolution at the time of the patient’s discharge to home.

## Introduction

Wounds, especially chronic wounds, can be clinically challenging to manage. Their presence poses physical, psychological, and even life-threatening problems to the patients, and an economic burden of approximately 28.1 to 96.8 billion dollars annually to society, according to Medicare cost projections [1]. The care of patients with wounds does not fall under a single specialty but rather is spread across multiple specialties and medical personnel, ranging from physicians to nurses. This lack of a unified specialty practice makes it challenging to provide patients with the highest quality, evidence-based practice for the management of complex wounds. While an attempt to remedy this situation has seen the creation of wound care centers in clinics and hospitals, the care of patients with wounds in skilled nursing facilities largely falls under the responsibility of the nurse, either a registered nurse or licensed vocational nurse who may not be equipped with the extensive knowledge and expertise to handle complex wounds. This can result in increased cost of care in the management of wounds in skilled nursing facilities, due to wounds taking longer to heal, or frequent hospital admissions of patients with wounds from skilled nursing facilities. To make things more complicated, patients in long-term care facilities usually suffer from multiple comorbidities, which can further complicate wound healing, thus requiring the expertise of highly trained physicians to provide both the highest quality of care for these patients and education for the entire facility on the science of wound care. This paper highlights the care of a patient in a skilled nursing facility who presented with a very complicated post-surgical abdominal wound, secondary to an infected post-surgical incision. The skilled nursing facility was very concerned about the size, depth, and nature of the wound and talks were underway for a possible transfer to a different long-term acute care facility or to the hospital for more specialized care. As a result of the weekly rounds of a specialized wound care physician at the facility, and a dedicated wound care nurse to execute the physician’s orders, the wound was adequately cared for and close to resolution at the time of the patient’s discharge to home.

## Case presentation

The patient was a morbidly obese 58-year-old female with a past medical history significant for hypertension, type two diabetes, and chronic nicotine use, who underwent surgical repair of a recurrent abdominal incisional hernia with lysis of adhesions. Her surgery was uneventful, and the patient was discharged home the following day. Two weeks later, the patient noted copious drainage and a foul odor from her surgical wound. She denied any fever or chills. She was evaluated at the local emergency room and noted to have a white count of 16,300 cells per microliter, with copious amounts of purulent drainage and a strong foul odor from her wound, which had become very necrotic. The patient was taken to the operating room by general surgery for wound debridement and washout and was started on intravenous vancomycin and Zosyn. She was subsequently discharged to a skilled nursing facility (SNF) for further post-acute care prior to discharging home. The staff at the SNF considered the wound too complicated to manage and opted to transfer the patient to a long-term acute care facility with more expertise. The wound care physician who conducts weekly rounds at the SNF was consulted for a second opinion and after further consideration, a decision was made to manage the wound at the SNF.

**Figure 1:**
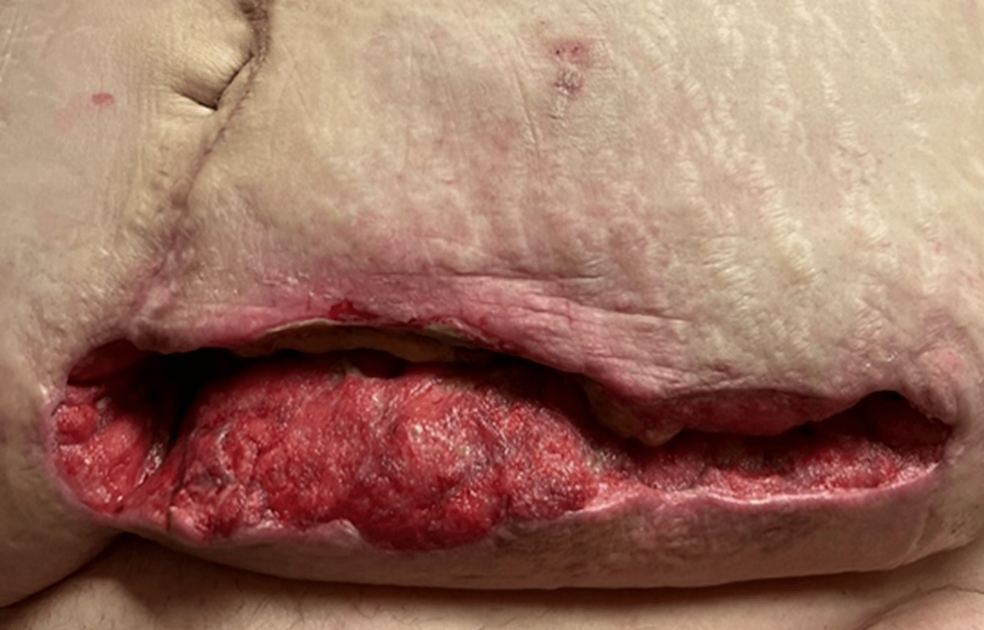
Post-surgical abdominal wound before wound consult

**Figure 2:**
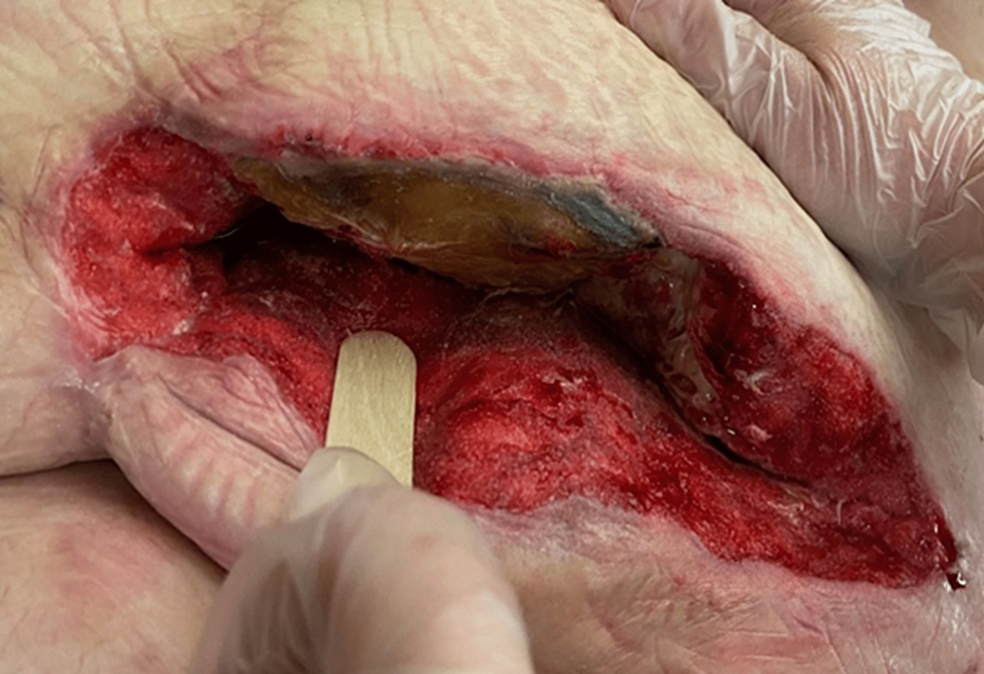
Post-surgical abdominal wound before wound consult

**Figure 3:**
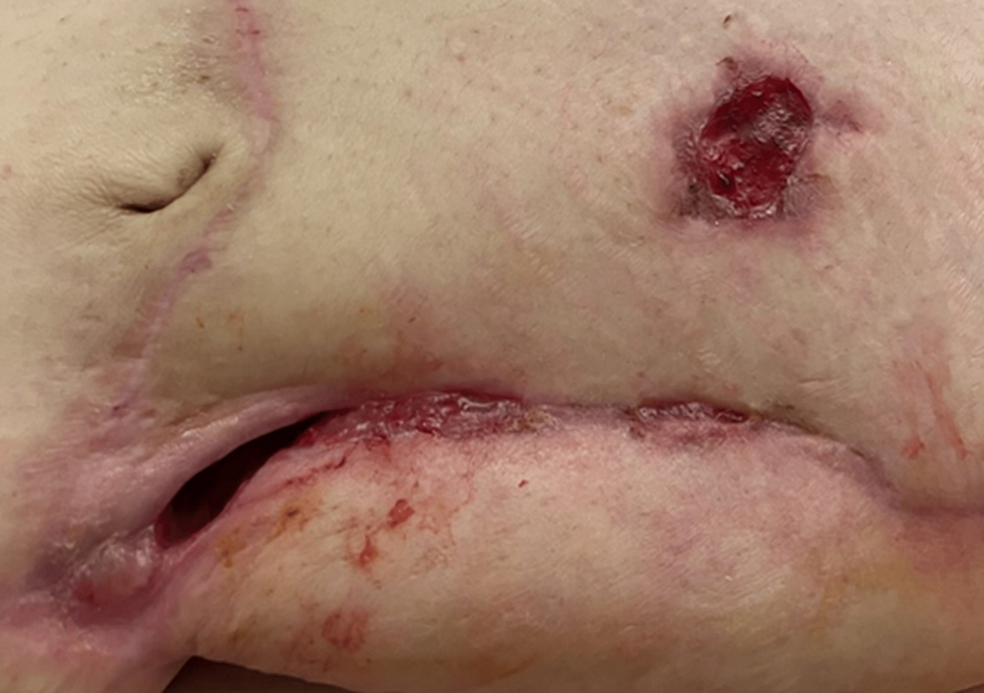
Post-surgical abdominal wound, before discharge from the skilled nursing facility, following treatment

**Figure 4:**
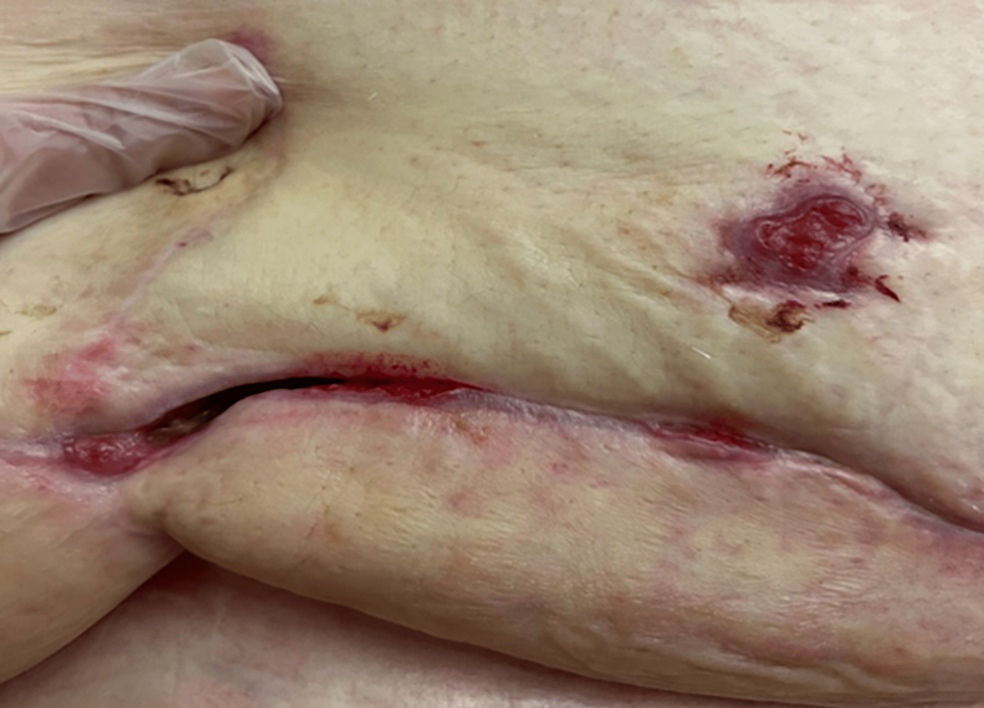
Post-surgical abdominal wound prior to discharge from the skilled nursing facility

Initial evaluation of the wound revealed a post-surgical abdominal wound, measuring 25 cm long, 7 cm wide, and 8 cm in depth (Figures 1, 2). The wound bed was pink, with 100% granulation tissue. The wound had heavy exudative drainage, and the nursing staff was struggling to keep up with dressing changes. Negative pressure wound therapy was ordered by the wound physician and four weeks later, the depth of the wound improved from 8 cm to 5 cm [2]. At the eight-week follow-up, with continued negative pressure wound therapy, the size of the wound had decreased to 17 cm in length and 2.5 cm in width, but some necrotic tissue build-up was noted on the wound bed. Weekly debridement of the wound was performed by the wound physician and ongoing treatment with negative pressure wound therapy was maintained three times a week. On the patient’s twelfth week of follow-up, the dimensions improved to 15 cm in length, 2 cm in width, and 2 cm in depth. At the patient’s request and on further re-evaluation of the wound, negative pressure wound therapy was discontinued and the wound physician ordered collagen powder, calcium alginates, and occlusive dry dressings for the wound, to be changed daily. Due to significant improvement in the patient’s wound and her physical health, the patient opted to be discharged home, and her final wound evaluation revealed a length of 5 cm, width of 2 cm, and depth of 2 cm (Figures 3, 4). Skilled home health care was ordered to continue managing the patient’s wound at home.

## Discussion

Skilled nursing facilities play a significant role in the care of patients recovering from a recent hospital stay or long-term care patients who can no longer be adequately cared for at home. The quality of care received by these patients plays a big role in determining their overall survival, hospital readmission rates, post-surgical infection rates, and many more. Unfortunately, these skilled nursing facilities are not staffed by highly specialized medical professionals and rely on nearby hospitals and specialty clinics to manage both acute and chronic medical conditions. This back-and-forth transportation not only costs money and resources but also places a physical and mental burden on the residents who are often frail and debilitated from multiple medical conditions. Bringing specialized medical care to residents in skilled nursing facilities will not simply eliminate the issue of convenience but will greatly elevate the quality of care for the residents. One such area that has benefited tremendously from the medical expertise of highly trained physicians is wound care. Wounds in nursing homes can be ubiquitous, given the older population with multiple chronic medical conditions [3,4]. Their management traditionally falls under the responsibility of a nurse, tasked with daily dressing changes, but chronic wounds can become non-healing due to a myriad of reasons, requiring specialized medical knowledge and training to adequately diagnose and treat these chronic wounds [5]. Without such expertise, nurses are forced to send patients to the hospital or clinic for perceived wound-related problems.

This case highlights the importance and necessity of specialized wound care physicians in skilled nursing facilities. The patient presented with a post-surgical wound whose size was very concerning to the facility. Also, communication from the discharging hospital was very poor, and offered the wound care nurse no clear instructions on how to manage the wound. The options for such wounds are to either send the patient to a post-acute care facility with the ability to manage such wounds or keep the wound in-house and make frequent trips to the local hospital or clinic for any wound-related concerns. These issues were avoided, and the patient was managed entirely in-house with very good outcomes with the help of a wound care physician performing weekly rounds and monitoring the progress of the wound. Treatment choices were guided by sound clinical practices and necrotic tissue was adequately debrided to reveal healthy tissue as needed. During her management, some parts of the wound became necrotic, excessive drainage was noted on some days with a foul odor, and the patient frequently reported increased pain from the wound. Recommendations for appropriate pain management and antibiotic coverage were made to provide a holistic approach to the patient’s wound care, and the wound care nurse was instructed to call the wound physician for all wound-related deterioration rather than direct hospital admission. There was no wound-related hospitalization while under the care of the wound physician at the SNF.

## Conclusions

Managing complex chronic wounds can be very challenging, especially in skilled nursing facilities. Poor management can add to the pain and suffering of the patient, with the potential for life-threatening wound deterioration, and an overall increase in the cost of wound care. Bringing specialized wound care physicians into skilled nursing facilities has the potential to remedy all these. Due to the challenges involved in managing patients in these facilities, more research is needed to explore innovative ways of providing high-quality wound care in these facilities, and policymakers should work with healthcare providers to identify opportunities to improve care through legislation.
